# Effect of Different Antipsychotic Drugs on Short-Term Mortality in Stroke Patients

**DOI:** 10.1097/MD.0000000000000170

**Published:** 2014-11-28

**Authors:** Jen-Yu Wang, Cheng-Yi Wang, Chen-Hui Tan, Ting-Ting Chao, Yung-Sung Huang, Ching-Chih Lee

**Affiliations:** From the Department of Internal Medicine (J-YW,C-YW, C-HT) and Medical Research Center (T-TC), Cardinal Tien Hospital, School of Medicine, Fu-Jen Catholic University, New Taipei City; Department of Neurology (Y-SH) and Department of Otolaryngology (C-CL), Dalin Tzu Chi Hospital, Buddhist Tzu Chi Medical Foundation, Chiayi; and School of Medicine (CCL), Tzu Chi University, Hualien, Taiwan.

## Abstract

The safety, tolerability, and efficacy data for antipsychotic drugs used in the acute phase of stroke are limited. The primary aim of this study was to examine the effectiveness and safety of typical and atypical antipsychotics on acute ischemic stroke mortality.

This observational study was conducted in a retrospective cohort of patients selected from the 2010–2011 National Health Research Institute database in Taiwan. Patients were tracked for 1 month from the time of their first hospitalization for acute ischemic stroke. A nested case–control analysis was used to estimate the odds ratio (OR) of 30-day mortality associated with antipsychotic drug, adjusted for age, gender, disease severity, and comorbidities.

The study cohort included 47,225 subjects with ischemic stroke, including 9445 mortality cases and 37,780 matched controls. After adjustment for the covariates, antipsychotics users before ischemic stroke are associated with a 73% decrease in the rate of mortality (OR 0.27; 95% CI 0.23–0.31). After ischemic stroke, the use of antipsychotics is associated with 87% decrease in the rate of mortality (OR 0.13; 95% CI 0.1–0.16). The users of conventional antipsychotics are associated with a 78% decrease in the rate of mortality (OR 0.22; 95% CI 0.18–0.26). The users of atypical antipsychotics are also associated with a 86% decrease in the rate of mortality (OR 0.14; 95% CI 0.12–0.17).

We found that 1-month mortality among acute stroke patients treated with antipsychotics is significantly lower. The benefit on lower mortality was found not only among ischemic stroke patients who had received antipsychotics previously but also among patients who start antipsychotics after their stroke.

## INTRODUCTION

Diagnosis and treatment of acute stroke have advanced over the past 2 decades, but morbidity and mortality after stroke are still high. Patients who have had stroke are at significant risk for medical complications, neurological damage, and various psychiatric illnesses. Even if not always life-threatening, these complications can lead to prolonged hospitalization, delay in rehabilitation, poor functional outcomes, and increased costs of care.^1–5^ Acute neurological complications include brain edema, hemorrhagic transformation, recurrent stroke, seizure, and epilepsy.^4,6^ Psychiatric complications include depression, psychosis, confusion, and delirium.^5,7,8^ Many reviews have focused on medical complications and their management.^2,3,8^ There is a growing body of evidence to guide the management of the neurological complications^1,4^ but not the management of the psychiatric complications.

The incidence of delirium in the acute phase of stroke varies from 13% to 48%, depending on the study population and delirium definition.^9–12^ Given the longer hospitalization period and poorer prognosis, adequate treatment is important.^5,7^ Sedative and antipsychotic drugs are frequently used for treatment of delirium in acutely ill patients, although the evidence base for use of these medications to treat delirium is weak.^13–16^ The treatment recommendations for delirium after stroke are usually similar to those for delirium in patients with other diseases, because there have been no studies of delirium specifically in patients with acute stroke.^4^

Traditionally, haloperidol, a conventional antipsychotic, has been considered the treatment of choice.^13^ Because it may cause adverse extrapyramidal symptoms, haloperidol may be replaced by atypical antipsychotics such as risperidone, olanzapine, or quetiapine, which are as effective as haloperidol in controlling delirium.^13,16–18^ The safety, tolerability, and efficacy data for antipsychotic drugs used in the acute phase of stroke are limited. The primary aim of this study was to examine the effectiveness and effect of conventional and atypical antipsychotics on acute ischemic stroke mortality.

## METHODS

### Data Source

In 1995, Taiwan implemented a National Health Insurance (NHI) program that requires mandatory enrollment in the government-run, universal, single-payer insurance system and provides comprehensive benefits coverage. Currently, up to 99% of the 23 million residents of Taiwan receive medical care through the NHI program. Over 97% of the hospitals and clinics in Taiwan are contracted to provide health care services,^19^ which are reimbursed by the NHI Bureau, and all data related to these services are collected and input into the National Health Research Institute Database (NHIRD) by the National Health Research Institutes to provide a comprehensive record of medical care. The data consist of ambulatory care records, inpatient care records, and the registration files of the insured. The dataset includes all claims data from Taiwan's NHI program, which was implemented to pay for health care of all Taiwanese citizens.

This study was initiated after approval by the Institutional Review Board of the Buddhist Dalin Tzu Chi General Hospital, Chiayi, Taiwan. Since all identifying personal information was stripped from the secondary files before analysis, the review board waived the requirement for written informed consent from the patients involved.

### Study Design

**FIGURE 1:**
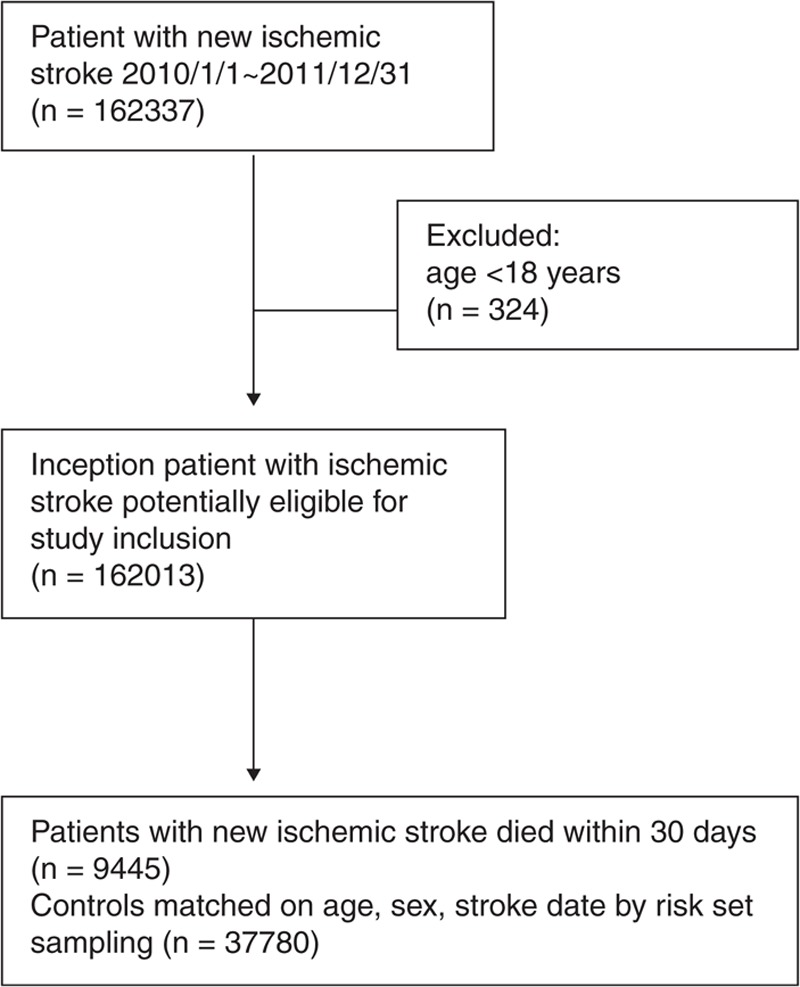
Flowchart of cohort formation.

The source population consisted of all patients with ischemic stroke between 2010 and 2011 (Figure 1). Inpatients ≥18 years of age diagnosed with stroke between 2010 and 2011 and with a discharge diagnosis identified by International Classification of Disease, 9th Revision, Clinical Modification (ICD-9-CM) codes 433–437 were recruited for the study. All patients with ischemic stroke in this cohort were followed from cohort entry until event (mortality) or censored developed. In view of the time-varying nature of antipsychotic drugs, a nested case–control analysis within the cohort was performed.

### Cases and Controls

Patients with ischemic stroke who died within 30 days after stroke were defined as cases. For each case, 4 controls who did not die within 30 days after stroke matched on the case's age (±3 years), gender, and stroke date (±3 days) were randomly selected.

### Exposure to Antipsychotics

All prescriptions of antipsychotics, conventional, atypical, or combined use, were identified 30-day before and after the index date. Patients were grouped into the following cohorts on the basis of exposure to antipsychotic medications. The antipsychotics used in this study included typical antipsychotics: chlorpromazine, thioridazine, levomepromazine, loxapine, perphenazine, trifluoperazine, haloperidol, fluphenazine, droperidol, zuclopenthixol, and prochlorperazine; and atypical antipsychotics: amisulpride, aripiprazole, clozapine, olanzapine, paliperidone, quetiapine, risperidone, sulpiride, ziprasidone, and zotepine. Patients who took typical antipsychotic were designated conventional antipsychotic users. Patients who took atypical antipsychotics were designated atypical antipsychotic users. Patients who took conventional and atypical antipsychotics were designated combined users; the remaining patients were designated antipsychotics nonusers. Within each antipsychotics group, subjects were further divided into 3 smaller groups based on total amount of pills use (ie, low, medium, and high dose).

### Comorbidities and Related Variables

We assessed patients’ comorbidities, including history of hypertension, diabetes, hyperlipidemia, atrial fibrillation, coronary artery disease, depression, dementia, schizophrenia, bipolar disorder, and post-stroke complications including pneumonia, urinary tract infection, deep vein thrombosis, acute coronary syndrome, upper gastrointestinal bleeding, seizure, and delirium. Because the NHIRD lacks information on the severity of stroke, we used intensive care unit (ICU) admission as a proxy for severity.

### Statistical Analysis

SPSS (version 15, SPSS Inc, Chicago, IL) was used for data analysis. Crude and adjusted odds ratios (ORs) of mortality cases associated with antipsychotics use with 95% confidence intervals (CIs) were estimated by multivariate logistic regression to account for the matching of cases and controls. The ORs were adjusted for patients’ characteristics (age, gender, and comorbidities), admission to an ICU, length of stay and current use, dose, and past use of antipsychotics. Comorbidities included hypertension, diabetes, hyperlipidemia, atrial fibrillation, coronary artery disease, depression, dementia, schizophrenia, bipolar disorder, and post-stroke complications including pneumonia, urinary tract infection, deep vein thrombosis, acute coronary syndrome, upper gastrointestinal bleeding, seizure, and delirium. A 2-sided *P* value (*P* < 0.05) was used to determine statistical significance.

## RESULTS

The study cohort included 47,225 subjects with ischemic stroke. Table 1 summarizes the characteristics of 9445 cases of mortality and 37,780 matched controls. The mortality cases and their matched controls were around 77 years of age and 60.8% were men. The mortality cases had more admitted to ICU and longer length of stay. The mortality cases had higher incidence of complications. Table 2 compares these characteristics among the mortality cases according to the use of antipsychotics, showing that antipsychotics users were more likely to have dementia and depression, but similar with respect to other comorbidities. Table 3 compares these characteristics among the matched controls according to the use of antipsychotics.

**TABLE 1:**
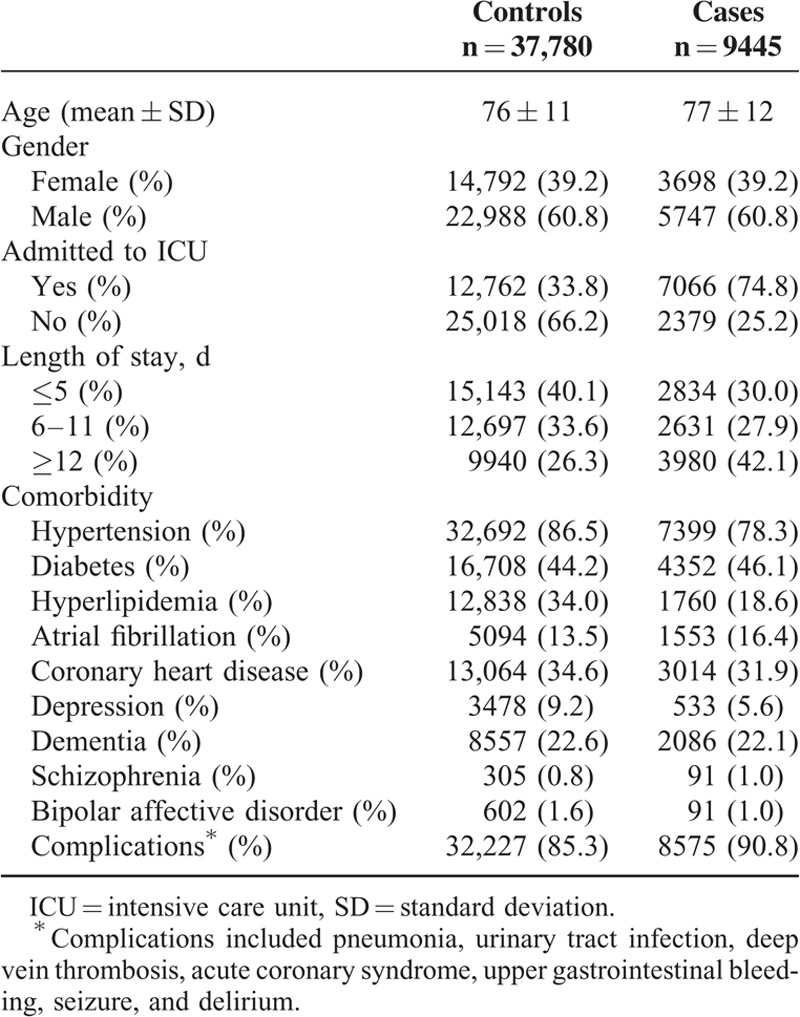
Characteristics of Mortality Cases and Their Matched Controls Selected From a Cohort of Patients With Ischemic Stroke

**TABLE 2:**
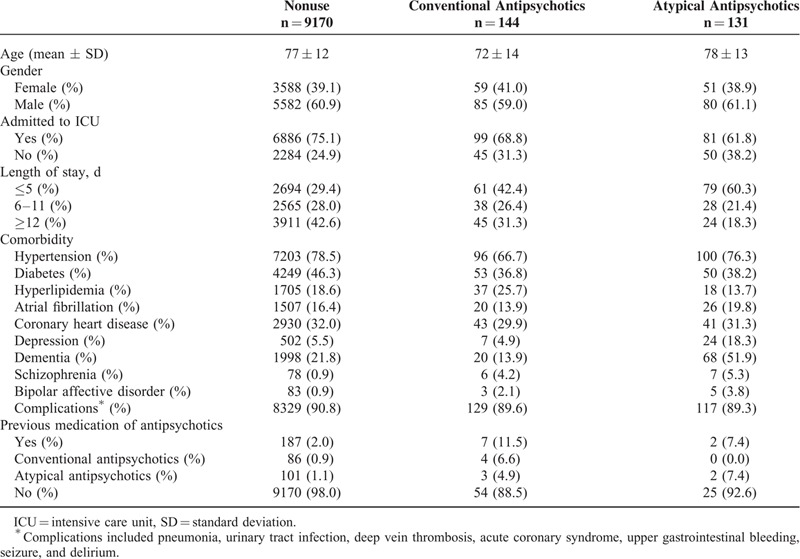
Characteristics of Mortality Cases Selected From Cohort of Patients With Ischemic Stroke, According to Current Use of Antipsychotic Medications After Stroke (n = 9445)

**TABLE 3:**
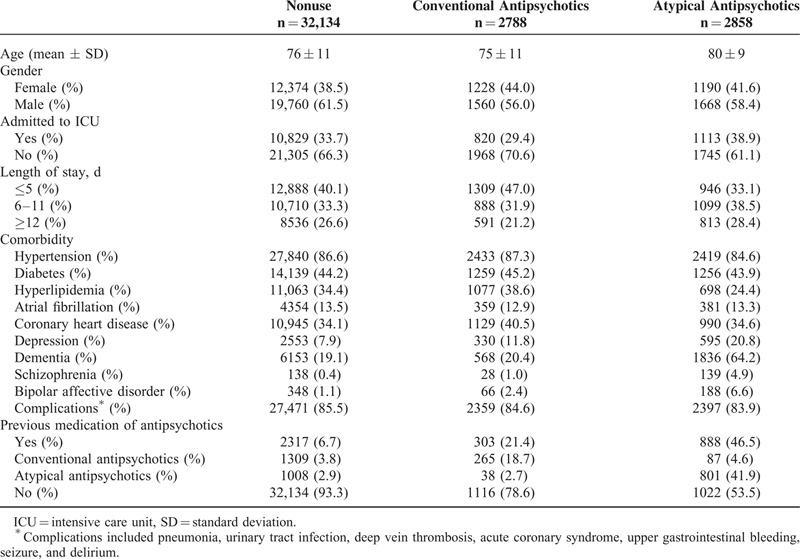
Characteristics of Matched Controls Selected From Cohort of Patients With Ischemic Stroke, According to Current Use of Antipsychotic Medications After Stroke (n = 37,780)

Table 4 shows that, after adjustment for differences in the covariates, antipsychotics users before ischemic stroke are associated with a 73% decrease in the rate of mortality (OR 0.27; 95% CI 0.23–0.31). After ischemic stroke, the use of antipsychotics is associated with 87% decrease in the rate of mortality (OR 0.13; 95% CI 0.1–0.16). The cumulative dose did not affect the benefit of antipsychotics use on ischemic stroke. The risk for mortality among users of conventional and atypical antipsychotics is shown in Table 5. The users of conventional antipsychotics are associated with a 78% decrease in the rate of mortality (OR 0.22; 95% CI 0.18–0.26). The users of atypical antipsychotics are also associated with a 86% decrease in the rate of mortality (OR 0.14; 95% CI 0.12–0.17).

**TABLE 4:**
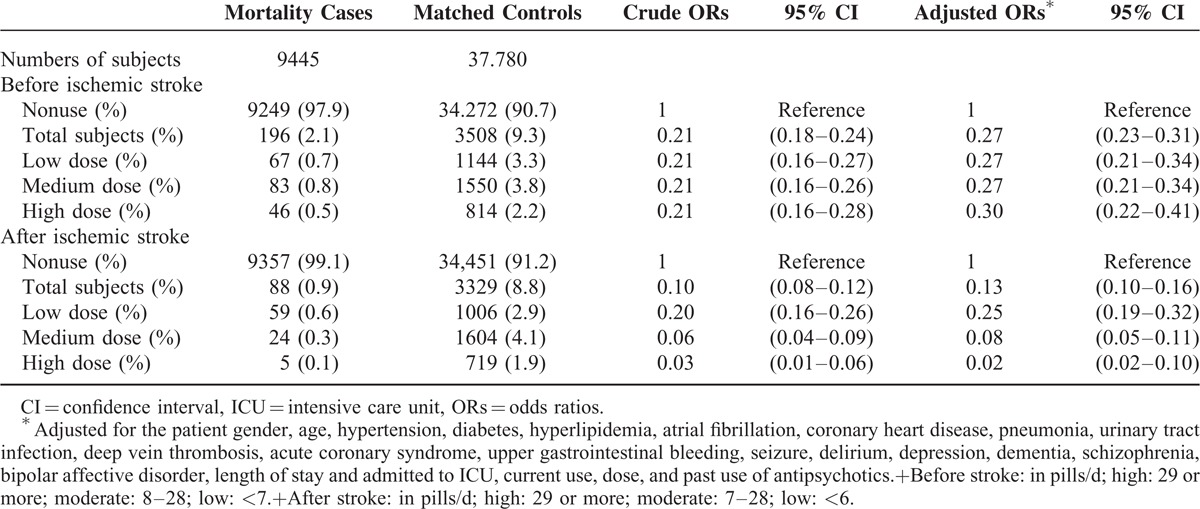
Crude and Adjusted Rate Ratios of Mortality Associated With Current Use, Dose, and Past Use of Antipsychotics

**TABLE 5:**
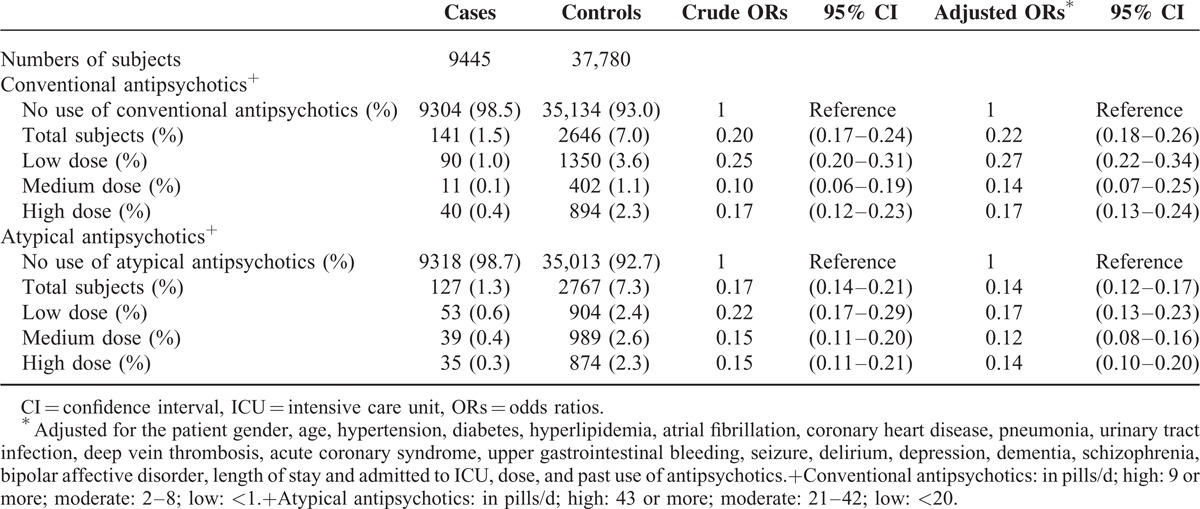
Crude and Adjusted Rate Ratios of Mortality Associated With Conventional Antipsychotics and Atypical Antipsychotics

## DISCUSSION

Using a large population-based cohort of 47,225 subjects with ischemic stroke, we found that both conventional and atypical antipsychotic drug users had lower 1-month mortality. The benefit on lower mortality was found not only among ischemic stroke patients who had received antipsychotics previously but also among patients who start antipsychotics after their stroke.

Atypical antipsychotics are used in the United States for the treatment of schizophrenia.^20,21^ Because of their superior efficacy, safety, and tolerability relative to conventional agents, atypical antipsychotics have become the mainstay of treatment in various neurological and psychiatric disorders.^22^ However, concerns have been raised that atypical antipsychotics may increase the risk of adverse events, including death and stroke.^23–26^ In contrast with these reports, studies from large observational administrative database studies suggest that atypical antipsychotics compared with conventional antipsychotics do not increase the risk of stroke.^27–29^ To date, the association between the use of atypical antipsychotics and the risk of stroke remains controversial. A recent investigation of the 90-day mortality rate after recent stroke among schizophrenia patients by Kang et al^30^ found lower mortality among patients who received antipsychotic medication before the stroke. Another study by Prior et al^31^ found that preadmission use of antipsychotics was associated with a higher risk of severe stroke, a longer duration of hospital stay, and a higher post-stroke mortality. The findings of this study suggest that, among past antipsychotics users, antipsychotics do not increase the risk of mortality for ischemic stroke. Racial and ethnic disparities in response to antipsychotics should be taken into consideration.

Many deleterious mechanisms of ischemic stroke have been proposed. In the ischemic brain, massive release of dopamine can amplify the neuronal damage caused by excitotoxicity and energy deprivation.^32,33^ Serotonin is also shown to modulate the postsynaptic effects of glutamate and lead to reduction of blood flow during cerebral ischemia.^34^ Atypical antipsychotics have been shown to have neuroprotective properties against cerebral ischemia in animal studies.^35,36^ The antidopaminergic and antiserotonergic effects of atypical antipsychotics could theoretically be beneficial by augmenting blood flow during ischemic stroke.^34^ Our study found that patients with acute ischemic stroke who received atypical antipsychotics had lower mortality, consistent with findings in animal models. Therefore, we propose that the observed lower mortality may be due to the neuroprotective effect of atypical antipsychotics.

In this study, we also showed that it is safe to treat patients in the acute phase after ischemic stroke with conventional antipsychotics, such as haloperidol. Haloperidol may interfere with recovery after stroke and therefore should be avoided if possible.^37^ In the practice guideline by the American Psychiatric Association for the treatment of delirium, haloperidol is the first choice,^38^ despite its anticholinergic side effects, a risk factor for delirium.^39^

Our study had several NHIRD database-related limitations. First, the diagnoses of stroke and any other comorbid conditions were dependent on ICD codes from the NHIRD database. The NHI Bureau of Taiwan, however, has made every effort to verify the accuracy of diagnosis by conducting random chart reviews and patient interviews. The accuracy of the NHIRD in recording ischemic stroke diagnoses is up to 95%.^40^ Second, an additional limitation of the NHIRD database was its lack of information on stroke severity. We used ICU admission and length of stay as proxies for severity. Third, the limitations of this study are those of observational studies, with the potential for uncontrolled confounding by prescription indication being the most troublesome. It is not possible to completely exclude prescription indication bias. Among both cases and controls, antipsychotics users were more likely to have dementia and depression. Nonetheless, given the magnitude and statistical significance of the observed effects in this study, these limitations were unlikely to have affected our results. In fact, the strengths of our study are that it was a nationwide population-based study, with near complete follow-up information on the whole study population (99%), and that the dataset was routinely monitored for diagnostic accuracy by the NHI Bureau of Taiwan.

## CONCLUSION

We found that 1-month mortality among acute stroke patients treated with antipsychotics is significantly lower. The benefit on lower mortality was found not only among ischemic stroke patients who had received antipsychotics previously but also among patients who start antipsychotics after their stroke. Well-designed studies focusing on antipsychotics use in acute stroke patients are needed to define optimal care.
